# Study on the effect of magnetic field treatment of newly isolated *Paenibacillus* sp.

**DOI:** 10.1186/s40529-015-0083-9

**Published:** 2015-01-30

**Authors:** Jie Li, Yanli Yi, Xilei Cheng, Dageng Zhang, Muhammad Irfan

**Affiliations:** 1 Key Laboratory of Preservation of Northeast Cultivated Land, Ministry of Agriculture, National Engineering Laboratory for Efficient Utilization of Soil and Fertilizer, College of Land and Enviroment, Shenyang Agricultural University, 120 dongling road, Shenyang, 110866 Liaoning China; 2 Liaoning Academy of Environmental Sciences, Shenyang, China; 3 Bioscience and Biotechnology College, Shenyang Agricultural University, 120 dongling road, Shenyang, 110866 China

**Keywords:** Magnetic treatment, Paenibaccilus sp, Catalase, Peroxidase, Superoxide dismutase

## Abstract

**Background:**

Symbiotic nitrogen fixation in plants occurs in roots with the help of some bacteria which help in soil nitrogen fertility management. Isolation of significant environment friendly bacteria for nitrogen fixation is very important to enhance yield in plants.

**Results:**

In this study effect of different magnetic field intensity and treatment time was studied on the morphology, physiology and nitrogen fixing capacity of newly isolated *Paenibaccilus* sp. from brown soil. The bacterium was identified by 16S rDNA sequence having highest similarity (99%) with *Paenibacillus* sp as revealed by BLAST. Different magnetic intensities such as 100mT, 300mT and 500mT were applied with processing time of 0, 5, 10, 20 and 30 minutes. Of all these treatment 300mT with processing time of 10 minutes was found to be most suitable treatment. Results revealed that magnetic treatment improve the growth rate with shorter generation time leading to increased enzyme activities (catalase, peroxidase and superoxide dismutase) and nitrogen fixing efficiencies. High magnetic field intensity (500mT) caused ruptured cell morphology and decreased enzyme activities which lead to less nitrogen fixation.

**Conclusion:**

It is concluded that appropriate magnetic field intensity and treatment time play a vital role in the growth of soil bacteria which increases the nitrogen fixing ability which affects the yield of plant. These results were very helpful in future breading programs to enhance the yield of soybean.

**Electronic supplementary material:**

The online version of this article (doi:10.1186/s40529-015-0083-9) contains supplementary material, which is available to authorized users.

## Background

*Paenibacillus* genus of bacteria was first included in Bacillus genus and then reclassified to a separate genus in 1993 (Ash et al. [1993]). These bacteria found in variety of environments like soil, water, forage, rhizosphere, insect larvae, vegetable matter and in clinical samples (McSpadden Gardener [2004], (Montes et al. [2004]; Ouyang et al. [2008]; Lal & Tabacchioni [2009])). These bacteria are of prime importance in agriculture for nitrogen fixation and industrial importance due to production of antibiotics and enzymes (Mavingui & Heulin [1994]; Von der Weid et al. [2003]). These bacteria produce plant growth hormones, suppress phytopathogens and solubilize organic phosphate (Mavingui & Heulin [1994]; Lebuhn et al. [1997]; Pires & Seldin [1997]).

Nitrogen is very essential nutrient for the growth of plants. So, these bacteria fix nitrogen from the air and provide this nitrogen to plants in the form of ammonium ions or other nitrogenous compounds essential for growth. From this symbiotic association, plant provides some organic compounds synthesized from photosynthesis (Sawada et al. [2003]). These bacteria not only fix the nitrogen but also enrich the soil fertility, increase plant production, and improve the quality, degrade organic pollutants and production of vitamin B series compounds (Sierra et al. [1999]; Agus et al. [2000]). The nitrogen deficiency was recovered by these rhizobia (Fisher & Long [1992]). In this process, plant produced some reactive oxygen species including the hydrogen peroxide and hydroxyl radicals and superoxide anion by defence reaction (Lamb & Dixon [1997]; Santos et al. [2001]). So it was necessary to study the rhizobia catalase, peroxidase and superoxide dismutase active changes.

A lot of research showed that the magnetic treatments have certain stimulative effect on crop production and development and, it also affect the genetic quality of seeds ((Zhu et al. [1996]; Liu et al. [1996]; Yan et al. [1997]; He et al. [1999]; Mao et al. [2002]); Jia et al. [2002]; (Liu et al. [2003])). Enzyme as protein with catalytic activity has an important role in the life process, and as a catalyst it was increasingly being attention (Cheng et al. [2007]). Magnetic field on the influence of the enzyme activity has been reported (He et al. [1998]; Li et al. [2007]; Hua et al. [2008]), and this area now attracts more and more people’s attention, but most of these studies focused on animals, plants and very little research on bacteria. So this study was aimed to check the effect of magnetic field on soybean rhizobia isolated from brown soil and their enzyme activities (peroxidase, catalase and superoxide dismutase) under the influence various intensity of magnetic treatment.

## Methods

### Materials

The Brown soil samples were collected from Shenyang Agriculture University, Shenyang Liaoning P.R. China. The samples were kept in sterile plastic bags and transferred aseptically to the lab.

### Isolation of *Paenibacillus*

The *Paenibacillus* sp. were isolated using standard procedures, and were purified by repeatedly streaking the bacteria on yeast extract-mannitol agar (YMA) medium (Vincent [1970]) and stored at 4°C.

### Molecular identification of *Paenibacillus*

Genomic DNA of the newly isolated bacterial strain was extracted by method as described by Ausubel et al. ([1994]). The DNA was amplified using universal primers 27 F:5′ -GAGAGTTTGATCCTGGCTCAG-3′ and 1492R:5′ -GGYTACCTTGTTACGACTT-3′. PCR reactions were performed in 50 l volume containing 1 μL template DNA, 4 μL MgCl_2_ (25 mmol/L), 5 μL 10× PCR buffer (Mg^2+^free), 4 μL dNTP(10 mmol/L), 1 μL of each primer (10 μmol/L), 0.5 μL of TaqDNA polymerase (5u/μL) and 33.5 μL ddH_2_O. PCR amplification conditions as follows: Initial denaturation at 94°C for 5 min followed by 30 cycles of denaturation at 94°C for 30 s, annealing at 55°C for 30 s and extension 72°C for 1 min, final extension at 72°C for 10 min. Amplification products were separated by 1 · 0% agarose gel electrophoresis and visualized under UV light after staining with ethidium bromide. The amplified 16S rRNA gene was sequenced using ABI 3730xl DNA Analyzer (Applied Biosystems, USA). The sequences were identified based on similarity using the Basic Local Alignment Search Tool (BLAST) program National Centre for Biotechnology Information (NCBI) online standard (http://www.ncbi.nlm.nih.gov/).

### Magnetic treatment of soil

The soil was treated by magnetic field in 100 mT, 300 mT and 500 mT for 5 min, 10 min, 20 min and 30 min respectively. Soybean was planted in the treated soil samples using phosphate and potash fertilizers (75 mg kg^−1^P_2_O_5_;75 mg kg^−1^ K_2_O). After harvestation the plants and soil was used to determine the soybean nodulation and nitrogen fixation capacities.

### Magnetic treatment of *Paenibacillus* sp.

The *Paenibacillus* sp. was inoculated in 100 mL of YMA medium, incubated at 28°C for 36 h with agitation speed 200 rpm. The cell growth was measured by taking OD at 520 nm. After the cell growth, 25 mL of *Paenibacillus* sp. cell suspension was taken in a test tube and treated it with different magnetic fields like 100, 300 and 500 mT with different time period such as 0, 5, 10, 20 and 30 min. Each experiment was conducted in triplicates and *Paenibacillus* sp. without magnetic treatment was taken as control.

### Enzyme assay

The *Paenibacillus* sp. broth was centrifuge at 5000 × g, 4°C for 10 min. After centrifugation the supernatant was discarded and the pellet was suspended in 50 mmol L^−1^ phosphate buffer (pH 7.0) and then subjected to sonication. The homogenate solution was centrifuged for 10 min at 10000 × g, 4°C. After centrifugation, the supernatant was used for determination of peroxidase (POD), superoxidase dismutase (SOD) and catalase (CAT) activities. Catalase activity was assay of hydrogen peroxide based on the formation of its stable complex with ammonium molkbdate and the OD was measured at 405 nm (Fang et al. [2004]). One unit of catalase activity was defined as the decomposition of 1 μ mol of hydrogen peroxide per minute under standard assay conditions. Peroxidase activity was determined by hydrogen peroxide-dependent oxidation of guaiacol. Samples were mixed with guaiacol solution (20 mmol/L guaiacol in 0.1 mol/L phosphate buffer (pH 6.8) and 0.03% (v/w) hydrogen peroxide) (Bergmeger et al. [1983]). Increase in absorbance at 470 nm was recorded using UV-visible spectrophotometer. One unit of POD activity was defined as the change in absorbance of 0.01 per minute at room temperature. Total SOD activity was assayed by the inhibition of the photochemical reduction of pyrogallol (PAPG) by following the photo reduction of nitroblue tetrazolium (Cai et al. [2006]). One unit of SOD activity was defined as amount of enzyme producing a 50% suppression of PAPG reduction. All the Enzyme specific activity is expressed as U/ml.

### Total nitrogen determination

Total plant nitrogen (N) concentration was analysed with Kjeldahl determination and colorimetric method as described by Baethgen and Alley (Baethgen & Alley [1989]). Nitrogen fixed was calculated as the total plant nitrogen content at harvest, minus the total nitrogen content at the start of the treatments.

### Statistical analysis

The data obtained after experimentation was statistically evaluated using ANOVA at significance level of p < 0.05 by using computer based programme SPSS.

## Results and discussion

### Molecular identification of *Paenibacillus* sp.

**Figure 1:**
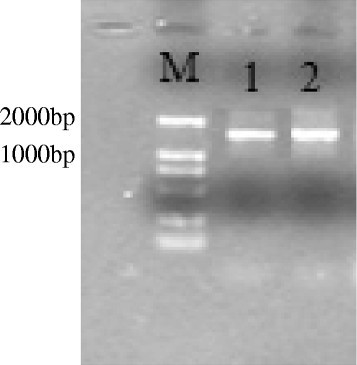
**Amplified product of 16S rDNA from newly isolated bacterial species.**

**Figure 2:**
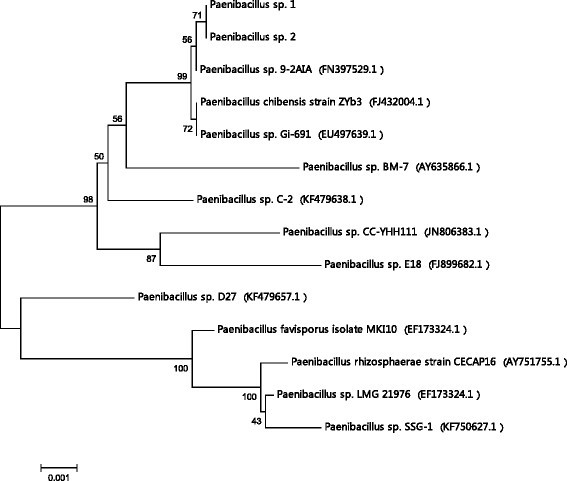
**Neighbor-joining phylogenetic analysis of 16S rDNA sequences (1442 bp) of newly isolated** ***Paenibacillus*** **sp. compared with the sequence of standard strains.** The bar represents a 0.002% of nucleotide variance.

The newly isolated bacterial strain was identified by molecular techniques using 16S rDNA sequencing. Product of 1442 bp was obtained after PCR amplification (Figure 1). The sequencing result was compared by BLAST for homology analysis. The isolated strain had 99% similarity with *Paenibacillus* sp. 9-2AIA (FN397529.1), *Paenibacillus* sp. Gi-691 (EU497639.1), *Paenibacillus chibensis strain ZYb3* (FJ432004.1), 98% similarity with *Paenibacillus sp. C-2* (KF479638.1), *Paenibacillus* sp. CC-YHH111 (JN806383.1), *Paenibacillus* sp. BM-7 (AY635866.1), 97% similarity with *Paenibacillus* sp. E18 (FJ899682.1), *Paenibacillus* sp. D27 (KF479657.1), *Paenibacillus favisporus* isolate MKI10 (EF173324.1) and 96% similarity with *Paenibacillus rhizosphaerae* strain CECAP16 (AY751755.1) and *Paenibacillus* sp. SSG-1 (KF750627.1) as shown in phylogenetic tree (Figure 2).

### Effect of magnetic field treated soil soybean nodular and nitrogen fixation

The soybean was planted in the brown soil treated with magnetic field, and the plants were harvested to measure the nodular situation and nitrogen content. The soybean was planted in the absence of nitrogen fertilizer and the difference in nitrogen content before and after treatment was used to compare the treatment effects. Thus we can use plant nitrogen to respond to the difference in the amount of nitrogen-fixing performance. Results shown in the Table 1 described that the soybean plant root dry weight, effective number of *Paenibacillus* sp. and total nitrogen content which treated by 100 mT, 300 mT magnetic field in plant were significantly higher as compared to control (CK). Results of this study was in agreement with Jing et al. (Jing et al. [1992]) who reported that appropriate intensity of magnetic field increased 4–5 time nitrogen fixing efficiency of the *Bradyrhizobium japonicum* 005. High treatment (500 mT) of magnetic field resulted decreased number of effective rhizobia thus leading to decreased total nitrogen content. This low nitrogen content leads to less growth because nitrogen is the main nutrition factor that influences the growth in plants (Marschner [1995]; Barker & Bryson [2007]). Nitrate is inorganic nitrogen which is dominant in agricultural soils and present in three or more times higher than natural soils (Hagedorn et al. [2001]; Owen & Jones [2001]).

**Table 1:** **Effect of magnetic field treated soil on soybean nodular and nitrogen content**

Treatment		Bacterial dry weight		Effective number of root nodule		Total nitrogen	
Magnetic field (mT)	Time (min)	Weight (g dry wt pl^−1^)	Percentage change (%)	Amount (No./plant)	Percentage change (%)	Content (mg/g)	Percentage change (%)
Control (CK)		0.06^h^	-	7 ± 1.23	-	3.23 ± 0.12	-
100	5	0.20^c^	233	24 ± 3.47	243	3.46 ± 0.13	7.12
	10	0.19^d^	217	18 ± 1.76	157.	3.51 ± 0.12	8.67
	20	0.17^e^	183	17 ± 1.84	143	3.35 ± 0.11	3.72
	30	0.15^f^	150	16 ± 1.34	129	3.25 ± 0.12	0.62
300	5	0.27^a^	350	37 ± 1.63	429	4.33 ± 0.17	34.06
	10	0.26^b^	333	28 ± 2.95	300	4.55 ± 0.15	40.87
	20	0.15^f^	150	24 ± 2.58	243	3.58 ± 0.13	10.84
	30	0.09^g^	50	13 ± 1.72	86	3.51 ± 0.16	8.67
500	5	0.04^j^	−33	7 ± 1.25	0	3.34 ± 0.13	3.41
	10	0.06^h^	0	7 ± 1.13	0	3.25 ± 0.12	0.62
	20	0.05^i^	−17	6 ± 1.13	−14	3.17 ± 0.11	−1.86
	30	0.05^i^	−17	7 ± 1.12	0	3.14 ± 0.13	−2.79

The different letters show significant difference (P < 0.05).

### Effect of magnetic treatment on generation time of *Paenibacillus* sp.

In order to check the effect of magnetic field on *Paenibacillus* sp. morphology and physiology, all the effects were studied before and after treatment using various magnetic intensities and treatment time. After the magnetic field treatment, *Paenibacillus* sp. I and *Paenibacillus* sp. II quantity and generation time were changed. On magnetic treatment, the number of *Paenibacillus* sp. was increased as compared to control (CK). This effect was changed with respect to magnetic intensity and time duration. Results (Table 2) showed that magnetic intensity of 300 mT had strong effect on the population of *Paenibacillus* sp. The growth of *Paenibacillus* sp. was influenced by magnetic intensity and magnetic treatment time. By increasing the treatment time, number of *Paenibacillus* sp. was increased and further increase in treatment time resulted decline in *Paenibacillus* sp. growth. Of all the treatment time, 10 minutes of magnetic treatment gave better yield. The generation time of 100 mT, 300 mT magnetic field intensity treatments were shortened as compared to control (CK) while 500 mT magnetic field intensity treatments has no significant effect on the generation time as compared to control. These results indicated that magnetic field treatment significantly enhance the *Paenibacillus* sp. population in shorter time. Cheng and Yi (Cheng & Yi [2009]) reported that magnetic field intensity of 300 mT had a significant positive effect on the generation time of slow-growing rhizobium (USDA110) and fast-growing rhizobium (USDA191).

**Table 2:** **The magnetic treatment of soybean purification number and generation of rhizobium time influence**

Treatment		***Paenibacillus***sp. I		***Paenibacillus***sp. II	
Magnetic field (mT)	Time (min)	Number	Generation of***Paenibacillus***sp. (h)	Number	Generation of***Paenibacillus***sp. (h)
CK		42	10.0	40	3.9
100	5	65	8.6	75	3.0
	10	101	8.2	68	3.1
	20	79	8.4	62	3.3
	30	55	8.6	64	3.5
300	5	85	8.0	122	3.1
	10	127	8.0	83	2.8
	20	95	8.1	77	3.1
	30	66	8.1	81	3.1
500	5	94	9.6	115	3.8
	10	102	9.6	84	3.9
	20	86	9.8	82	3.9
	30	42	9.7	78	4.0

### Effect of magnetic treatment on morphology of the *Paenibacillus* sp.

**Figure 3:**
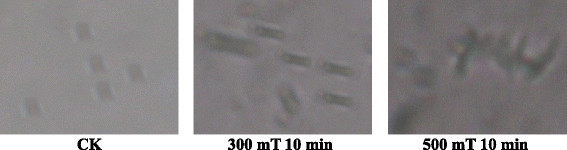
**Effects of magnetic fields on the morphology of** ***Paenibacillus*** **sp.**

**Figure 4:**
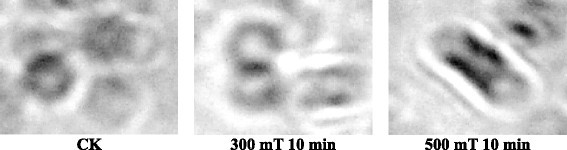
**Effect of magnetic fields on the morphology of** ***Paenibacillus*** **sp.II.**

The effect of magnetic field on morphology of *Paenibacillus* sp. was also studied as shown in the Figures 3 and 4. Results showed that morphology of *Paenibacillus* sp. was affected by magnetic treatment. Only 10 minutes of magnetic treatment time with intensity of 300 mT and 500 mT was studied. In 300 mT treatment the cell becomes thin and longer while in case of 500 mT treatment the cell membrane was ruptured and the cytoplasmic sap was released leading to the death of the *Paenibacillus* sp. These results revealed that suitable processing time with specific intensity can promote the growth of the *Paenibacillus* sp. but high and long time magnetic processing inhibited the growth thus leading to the death of *Paenibacillus* sp. Our findings were similar as reported by Fadel et al. (Fadel et al. [2003]). Various reports suggested that exposure of microorganisms to magnetic field caused changes in morphology and growth (Mohamed et al. [1997]; Gaafar et al. [2006]).

### Effect of magnetic field treatment on enzyme activity of *Paenibacillus* sp.

**Figure 5:**
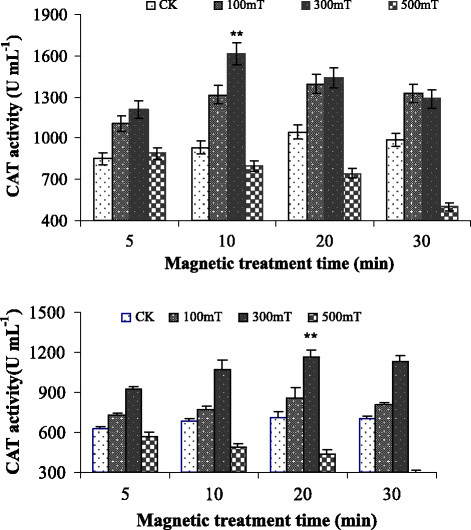
**Effect of magnetic fields on CAT activity of** ***Paenibacillus*** **sp.I (upper) and** ***Paenibacillus*** **sp.II (lower).** Error bars indicate SD among triplicates. Stars represent significance level at *P* > 0.05.

The influence of magnetic field was also checked on the catalase, peroxidase and superoxide dismutase activity of *Paenibacillus* sp. I and II. The catalases activity of *Paenibacillus* sp. I and II were enhanced by 300 mT intensity as shown in Figure 5. *Paenibacillus* sp. I showed more catalase activity as compared to *Paenibacillus* sp. II. The effective intensity and magnetic treatment time for *Paenibacillus* sp. I and II were 300 mT intensity for 10 and 20 minutes respectively. Both 100 mT and 300 mT increased the catalase activity while 500 mT decreased catalase activity as compared to control. The highest growth rate of *Paenibacillus* sp. I and II was 77% and 95% respectively. These results suggested that magnetic treatment time and intensity had strong influence on metabolic activity of *Paenibacillus* sp.

**Figure 6:**
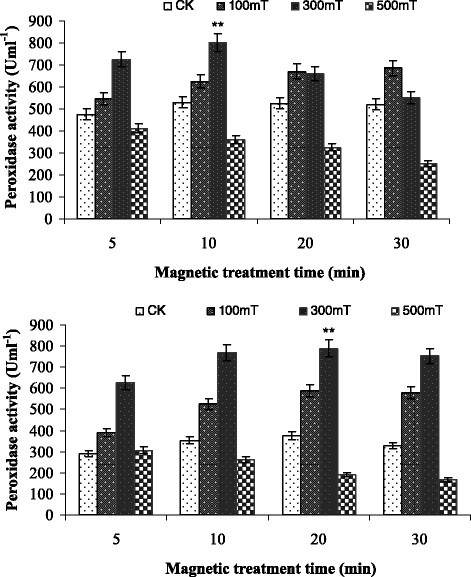
**Effect of magnetic field on Peroxidase activity of** ***Paenibacillus*** **sp.I (upper) and** ***Paenibacillus*** **sp.II (lower).** Error bars indicate SD among triplicates. Stars represent significance level at *P* > 0.05.

Figure 6 showed the influence of different magnetic field on peroxidase activity of *Paenibacillus* sp. The 100 and 300 mT magnetic field treatment have growth trend to the peroxidase activity of *Paenibacillus* sp. The highest growth rate of *Paenibacillus* sp. I and II was 68% and 203% in 10 min and 30 min magnetic treatment respectively. Both *Paenibacillus* sp. showed maximum peroxidase activity at 10 min of treatment time with intensity of 300 mT. This activity behavior is almost similar to that of catalase activity.

**Figure 7:**
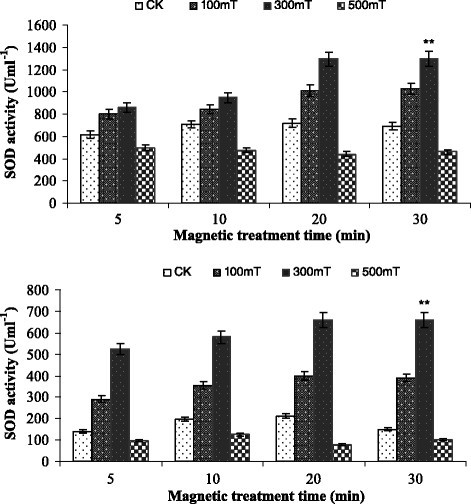
**Effect of magnetic fields on Superoxide dismutase activity (SOD) of** ***Paenibacillus*** **sp.I (upper) and** ***Paenibacillus*** **sp.II (lower).** Error bars indicate SD among triplicates. Stars represent significance level at *P* > 0.05.

Figure 7 depicted the effect of different magnetic field on superoxide dismutase activity from *Paenibacillus* sp. Superoxide dismutase activity was found maximum at 20 min of magnetic treatment with intensity of 300 mT. This enzyme activity also showed the same trend as the previous ones (catalase and peroxidase activity) but here increased treatment time resulted increased superoxide dismutase activity. In all the three enzyme activities (catalase, peroxidase and superoxide dismutase) magnetic field intensity of 500 mT inhibited the enzyme activity because this magnetic intensity ruptured the cell shape which leads to decline in enzyme activities. The growth rate at 300 mT magnetic treatment was 340% and 153% for *Paenibacillus* sp. I and II respectively. So this study was in good agreement with Liu et al. (Liu et al. [1996]) who reported that appropriate magnetic field intensity enhanced the activities of hydrogen peroxidases, invertases, amylases and phosphatases in the three tested soils. Another study also revealed that magnetic field enhances the catalase and superoxide dismutase activity isolated from the roots of soybean (Celik et al. [2009]).

## Conclusion

In conclusion the magnetic treatment significantly enhances the bacterial population with shorter generation time. This increased population of *Paenibacillus* sp. would increase the nitrogen fixing efficiency thus leading to greater yield. The enzyme activities were also increased under the influence of magnetic treatment. Increased magnetic field intensity and longer magnetic processing time resulted ruptured bacterial cell which leads to cell death, thus reduction in nitrogen fixation efficiency. To achieve the better yield, appropriate magnetic field intensity and magnetic processing time is very important for this whole process.

## Authors’ original submitted files for images

Below are the links to the authors’ original submitted files for images. Authors’ original file for figure 1 Authors’ original file for figure 2 Authors’ original file for figure 3 Authors’ original file for figure 4 Authors’ original file for figure 5 Authors’ original file for figure 6 Authors’ original file for figure 7
