# Supplementary figures and images for: Study on the effect of magnetic field treatment of newly isolated Paenibacillus sp
Source: Bot Stud. 2015 Jan 30;56:2. doi: 10.1186/s40529-015-0083-9 (PMC5430345; doi:10.1186/s40529-015-0083-9)

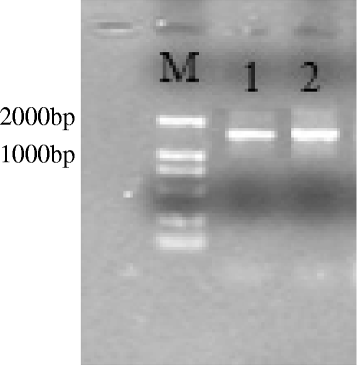

Supplement: Supplementary file 1 — Authors’ original file for figure 1 [file 40529_2015_83_MOESM1_ESM.gif]

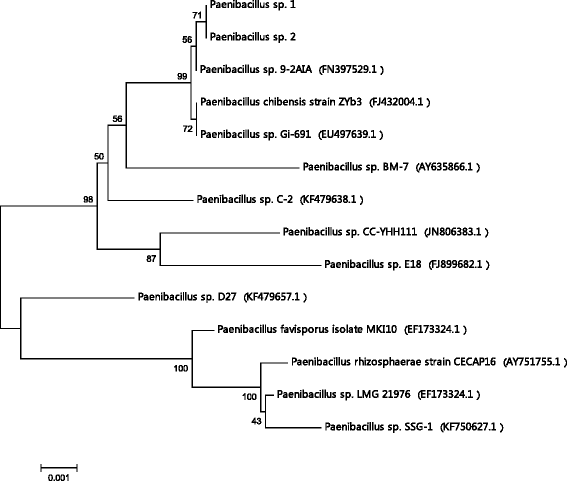

Supplement: Supplementary file 2 — Authors’ original file for figure 2 [file 40529_2015_83_MOESM2_ESM.gif]

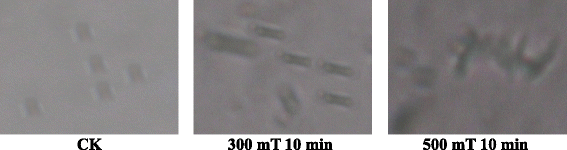

Supplement: Supplementary file 3 — Authors’ original file for figure 3 [file 40529_2015_83_MOESM3_ESM.gif]

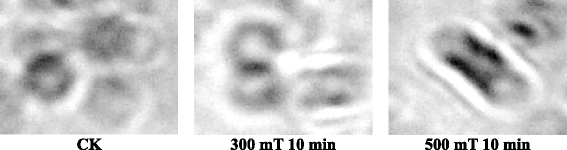

Supplement: Supplementary file 4 — Authors’ original file for figure 4 [file 40529_2015_83_MOESM4_ESM.gif]

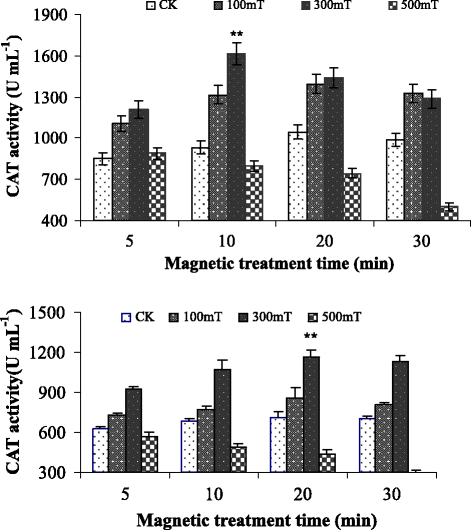

Supplement: Supplementary file 5 — Authors’ original file for figure 5 [file 40529_2015_83_MOESM5_ESM.gif]

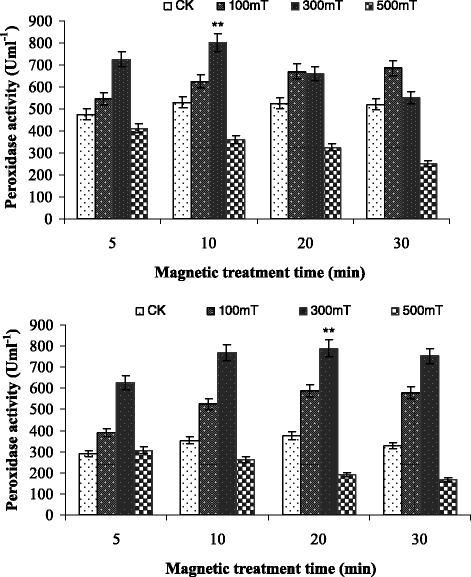

Supplement: Supplementary file 6 — Authors’ original file for figure 6 [file 40529_2015_83_MOESM6_ESM.gif]

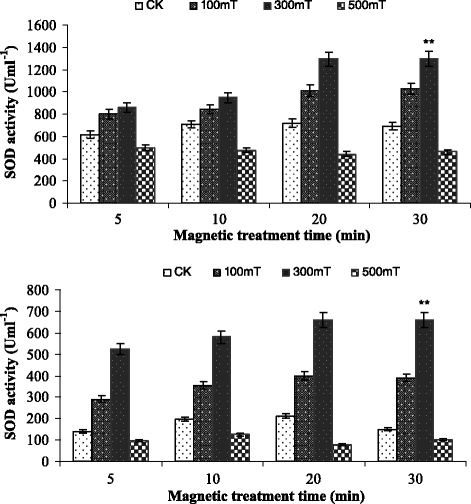

Supplement: Supplementary file 7 — Authors’ original file for figure 7 [file 40529_2015_83_MOESM7_ESM.gif]
